# Colorectal Leiomyosarcoma: Demographics Patterns, Treatment Characteristics, and Survival Analysis in the U.S. Population

**DOI:** 10.1007/s12029-024-01110-x

**Published:** 2024-08-27

**Authors:** Abdul Qahar Khan Yasinzai, Kue Tylor Lee, Imran Khan, Bisma Tareen, Amir Humza Sohail, Asif Iqbal, Israr Khan, Abdul Waheed, Bhavishya U. Ramamoorthy, Asad Ullah, Andrew M. Blakely

**Affiliations:** 1 University of Florida Health Cancer Center, Gainesville, FL 32608 USA; 2 Medical College of Georgia, Augusta, GA 30912 USA; 3 Department of Colorectal Surgery, Cleveland Clinic, Cleveland, OH 44195 USA; 4 Department of Medicine, Bolan Medical College, Quetta, 83700 Pakistan; 5 Department of Surgical Oncology, University of New Mexico, Albuquerque, NM USA; 6 Department of Medicine, Northeastern Health System, Tahlequah, OK USA; 7 Insight Hospital and Medical Center, Chicago, USA; 8 Department of Surgery, Baycare Health System, Clearwater, Fl USA; 9 Surgical Oncology Program, National Cancer Institute, Bethesda, MD 20892 USA; 10 Department of Pathology, Texas Tech University Health Sciences Center, Lubbock, TX 79430 USA

**Keywords:** Colorectal Leiomyosarcoma, SEER, Surgery

## Abstract

**Background:**

Colorectal leiomyosarcoma (CR-LMS) is a rare neoplasm arising from smooth muscle cells. It accounts for less than 0.1% of all colorectal malignancies. In this population-based study, we aim to understand the demographics, treatment characteristics, and pathologic factors associated with survival in CR-LMS.

**Methods:**

Data from the SEER Program (2000–2018) were analyzed using SEER*Stat and SPSS. Statistical methods included descriptive analysis, Kaplan–Meier survival curves, log-rank tests, and Cox proportional hazards regression to assess the impact of various factors on disease-specific and overall survival.

**Results:**

A total of 191 cases of CR-LMS were identified. Most patients were 60–69 years of age (median: 64 years) and Caucasian (78%). There was nearly the same distribution in sex (M**:**F ratio; 1**:**1.2). The overall 5-year observed survival was 50.3% (95% C.I., 46.3—54.2). The 5-year disease-specific survival (DSS) was 66.1% (95% C.I., 62.0—70.1). The 5-year overall survival after resection was 60.8% (95% C.I., 56.3—65.3). Multivariable analysis identified grades III and IV (p = 0.028) as negative predictors of overall survival. Regional spread and distant stage are negative predictors of overall survival (p < 0.01).

**Conclusion:**

Our data reveals that colorectal leiomyosarcoma (CR-LMS) often presents in patients around 64 years old with advanced stages and poor differentiation. Key adverse prognostic factors include older age, high tumor grade, large tumor size, and distant metastases, with surgical resection showing the best survival outcomes. To improve outcomes, further research and consolidation of data are essential for developing targeted therapies and comprehensive guidelines.

**Supplementary Information:**

The online version contains supplementary material available at 10.1007/s12029-024-01110-x.

## Introduction

Colorectal leiomyosarcoma (CR-LMS) arises from smooth muscle cells in the muscularis propria [1]. The condition is exceedingly rare, accounting for only 0.1% of colorectal cancers [2]. When symptomatic, patients with CR-LMS most commonly present with abdominal pain, hematochezia or melena, altered bowel habits, and/or tenesmus [3]. CT is the imaging modality of choice, as it provides invaluable information about the size and location of the primary tumor and detects regional/distant metastatic disease. On colonoscopy, LMS appears as an intraluminal, bulging, polypoid mass. A definitive diagnosis can only be made by pathologic analysis. Most CR-LMS are positive for desmin and caldesmin but lack CD34, CD117, and DOG1 expression, thus ruling out gastrointestinal stromal tumor (GIST) [4]. Surgical excision remains the mainstay of therapy, particularly when localized. The benefit of resection is partially diminished by frequent local recurrence despite achieving clear margins. Given the high rates of recurrence and associated mortality, radical excision is advocated even for localized disease to improve overall and disease-free survival [5]. These tumors are radioresistant, limiting adjunct treatment options [6].

The rarity of CR-LMS has led to a paucity of data, limiting insight into prognostic factors and the optimal therapeutic strategy. Much of the literature on CR-LMS is restricted to case series and small database studies. Furthermore, CR-LMS is associated with a progressive course and a higher rate of recurrence when compared to the stomach and small bowel LMS [7]. Unfortunately, some studies that report on outcomes in LMS include all GI sites in aggregate, limiting the ability to identify prognostic factors specific to CR-LMS versus LMS of other sites.

A population-based database study was chosen to identify a larger cohort of patients with CR-LMS. The primary objectives of this study are to assess the overall survival (OS), disease-specific survival (DSS), and overall survival by treatment modalities as it applies to CR-LMS, specifically. We also sought to identify the clinical, demographic, and pathological factors that influence survival outcomes in this rare yet aggressive malignancy.

## Materials and Methods

The Surveillance, Epidemiology, and End Results (SEER) Program was initiated by the National Cancer Institute in 1973 and covers approximately 28% of the United States population. SEER*Stat software (Version 8.4.1) was used to collect cancer patient data from 2000 to 2018 using the International Classification of Diseases for Oncology, Version 3 (ICD-O-3). Then, 18 registries from SEER were used to extract data. Patients were identified from the SEER registries using the ICD-O-3 topography codes C18.0, C18.2, C18.3, C18.4, C18.5, C18.6, C18.7, C18.8, C18.9, C19.9, and C20.9, as well as the ICD-O-3 morphology codes 8890/3, 8891/3, and 8893/3. In SEER, DSS is delineated by cause-specific survival (CSS). This data was then exported to Statistical Package for Social Sciences (SPSS), Version 28.0.0.0 (IBM Corp., Armonk, NY) for descriptive analysis.

Demographic and clinical data included age, race, sex, tumor grade, tumor size, lymph node status, metastasis, stage, and treatment received. Stage was identified with earlier versions of the AJCC guidelines and harmonized with the most recent AJCC guidelines (2016).

SPSS was used for statistical analyses of patient demographics, disease features, and factors impacting survival. Categorical data were expressed as proportions and analyzed using the Chi-squared test. Continuous variables were converted to categorical variables for subsequent analysis. Survival curves were generated using the Kaplan-Meyer method and compared using the log-rank test, with generation of p values, hazards ratios (H.R.), and 95% confidence intervals (C.I.).

Cox proportional hazards regression analysis was utilized to examine associations among demographic factors, tumor characteristics, and treatment modality with DSS and OS as the outcome measures. Multivariable Cox analyses were used to identify factors independently associated with survival; cases with unknown variables were censored from the multivariable analysis. A two-tailed p-value < 0.05 was considered significant.

## Results

**Fig. 1:**
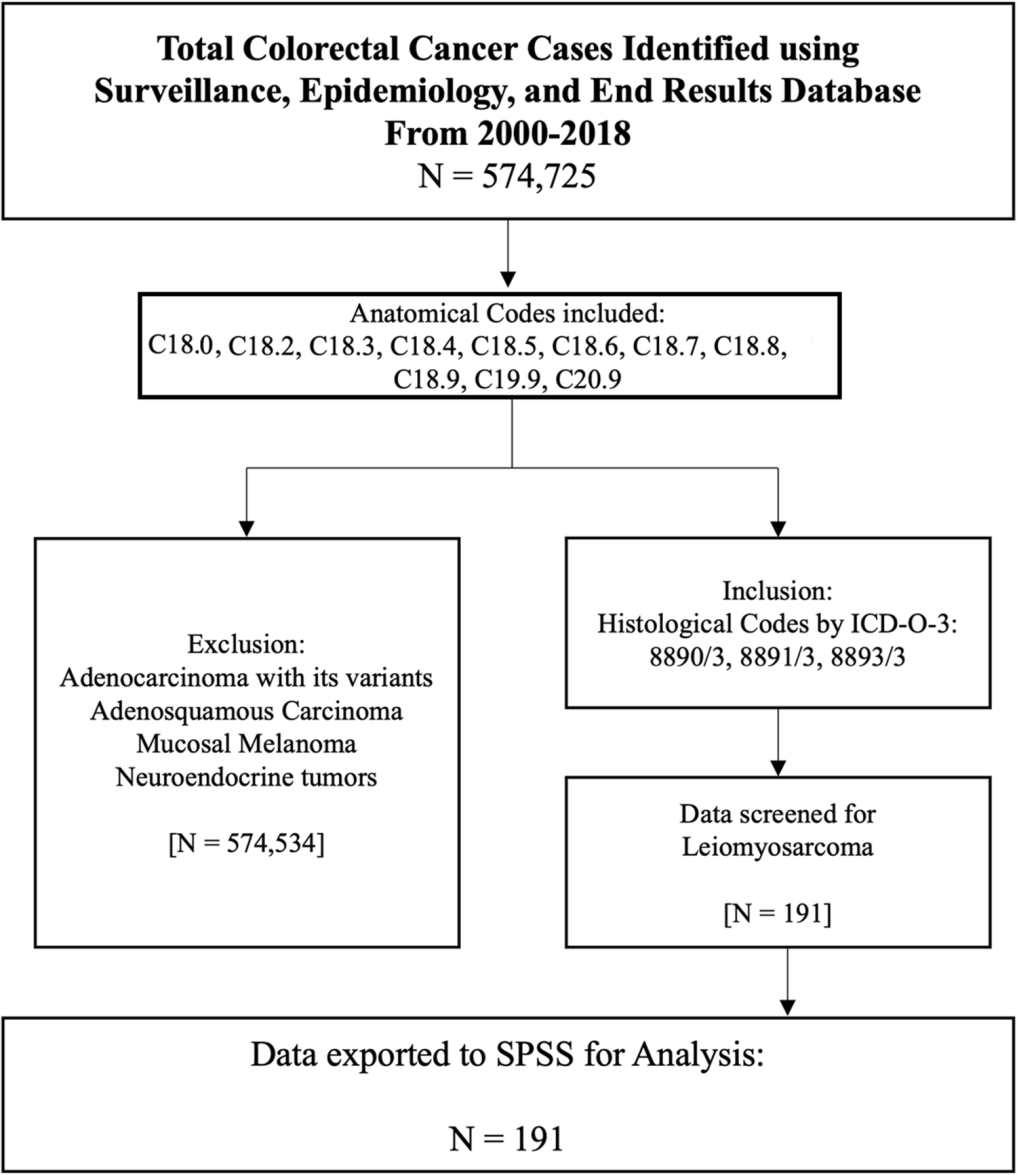
Flowchart for Colorectal Leiomyosarcoma

In this study, 191 cases of colorectal leiomyosarcoma were identified from 2000 – 2018 using the SEER database (Fig. 1).

### Demographic and Tumor Characteristics

The median age for the patients was found to be 64 years. The most common age groups were between the ages of 60–69 (23.0%) and 70–79 (23.0%). Females were the slight majority in this cohort with 54.5%, while 45.5% of the cases were male. In regard to race and ethnicity, the majority of patients were non-Hispanic white (78.0%), followed by non-Hispanic black (13.1%), non-Hispanic Asian or Pacific Islanders (7.3%), then non-Hispanic American Indian (0.5%).

When tumor grading was provided, most cases were poorly differentiated. Tumor sizes were known in 66.0% of cases. 7.9% of known cases were less than 2.0 cm, 26.2% were between 2.0 cm to 5.0 cm, and 65.9% were greater than 5.0 cm. The stage of the majority of cases were localized disease (45.5%) followed by regional (25.7%), then distant disease (15.7%) (Table 1). The tumor stage in 13.1% of cases was unknown. In 33.5% of the cases, the primary tumor site was the right colon (cecum to transverse colon), 35.6% in the left colon (splenic flexure to rectosigmoid junction), 23.6% in the rectum, and the primary site was unspecified in 5.2% cases. Lymph node status was known in 70.7% of cases. Of the statuses known, the majority of cases had positive lymph nodes (65.9%). While the site of distant metastases was unknown in the majority of cases, the most common site of metastasis was the liver (5.6%), followed by the lung (4.2%), and bone (1.1%) (Table 1).

**Table 1:** Demographic factors and tumor characteristics in colorectal leiomyosarcoma

Variable	Category	Total N = 191 N (%)
Patient Characteristics		
Age at Diagnosis, years	< 20	1 (0.5)
	20 – 29	3 (1.6)
	30 – 39	10 (5.2)
	40 – 49	21 (11.0)
	50 – 59	40 (20.9)
	60 – 69	44 (23.0)
	70 – 79	44 (23.0)
	≥ 80	28 (14.7)
Sex	Male	87 (45.5)
	Female	104 (54.5)
Race/Ethnicity	Unknown	2 (1.0)
	Non-Hispanic White	149 (78.0)
	Non-Hispanic Black	25 (13.1)
	Non-Hispanic Asian or Pacific Islander	14 (7.3)
	Non-Hispanic American Indian	1 (0.5)
Clinicopathologic Characteristics		
Size	< 2 cm	10 (5.2)
	2.0 to 5.0 cm	33 (17.3)
	> 5 cm	83 (43.5)
	Unknown	65 (34.0)
Grade	Low	10 (5.2)
	Moderate	21 (11.0)
	High	86 (45.0)
	Unknown	74 (38.7)
Primary Site	Right Colon	64 (33.5)
	Left Colon	68 (35.6)
	Rectum	45 (23.6)
	Not specified	10 (5.2)
Node Status	Negative	46 (24.1)
	Positive	89 (46.6)
	Unknown	56 (29.3)
Metastasis at Diagnosis	Unknown	181 (94.8)
	Known	10 (5.2)
Disease Stage	Localized	87 (45.5)
	Regional	49 (25.7)
	Distant	30 (15.7)
	Unknown	25 (13.1)

### Treatment Characteristics

**Fig. 2:**
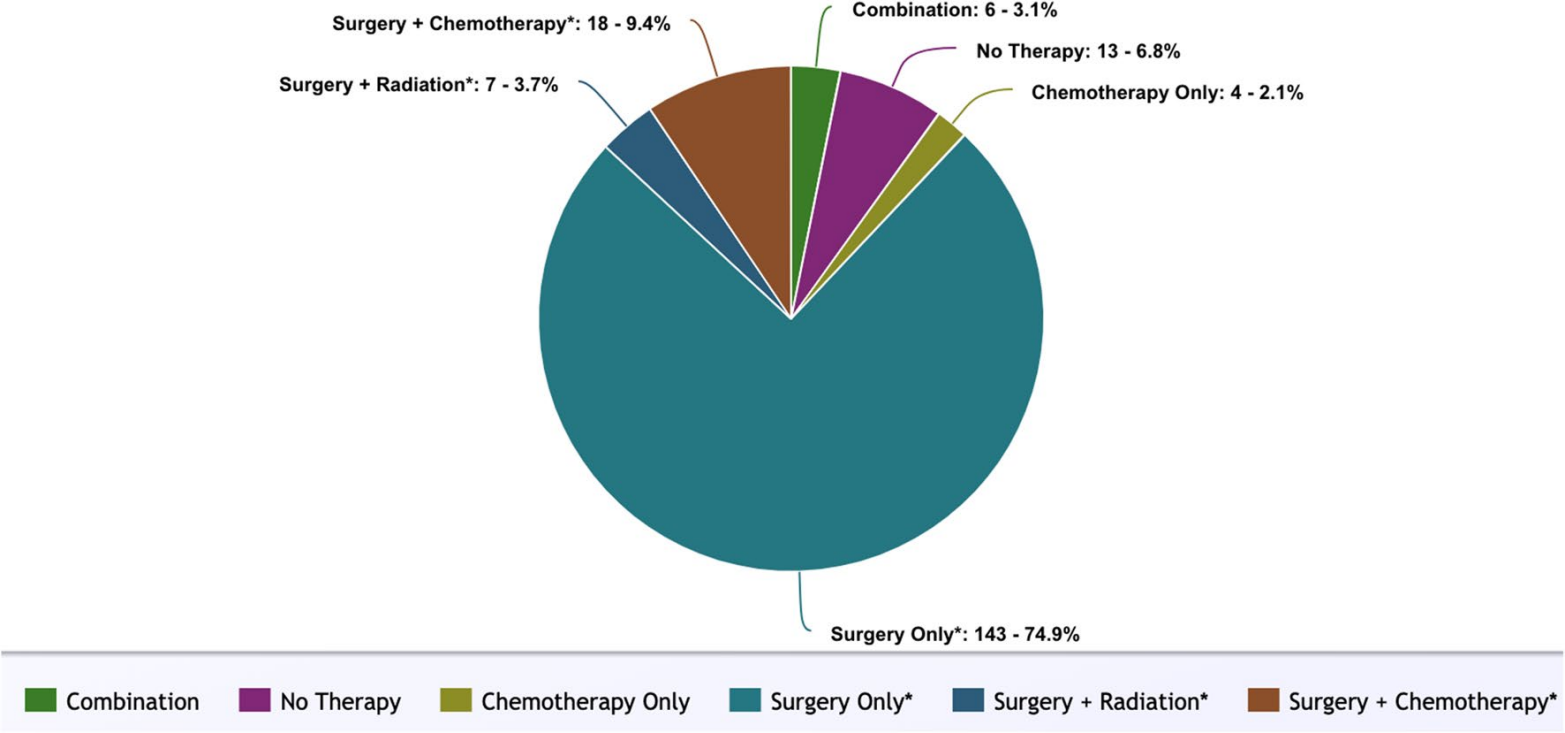
Pie chart describing treatment modalities

Of the total cases, the majority of cases in this cohort underwent surgery only (74.9%) Only 2.1% of cases had chemotherapy only (any systemic therapy). No cases had radiation only. 3.1% of cases underwent combination therapy (surgery, radiation, and chemotherapy). 6.8% of cases did not undergo any treatment (Fig. 2).

### Overall and Disease-Specific Survival by Treatment

**Fig. 3:**
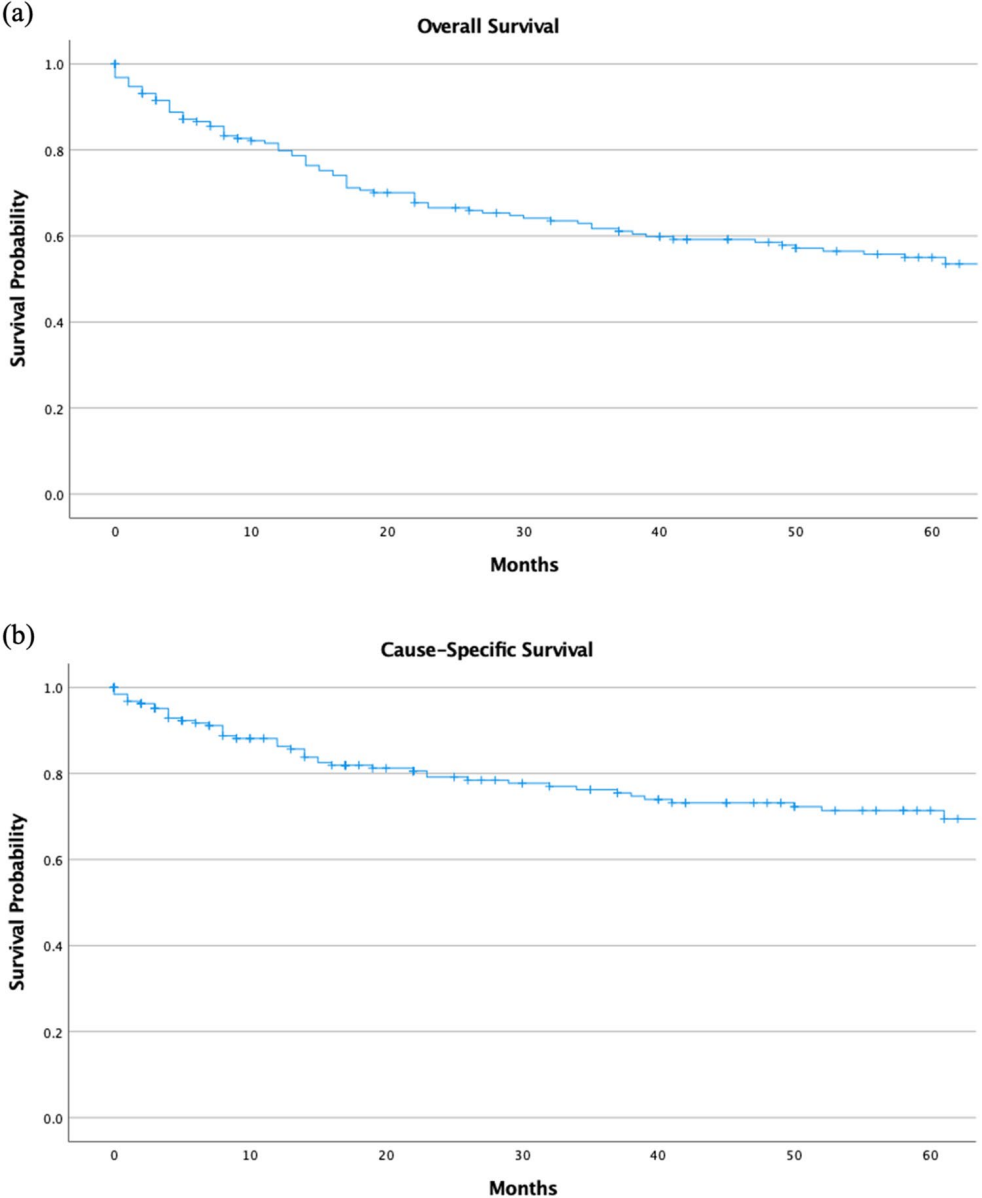
(**a**) Overall survival of Colorectal Leiomyosarcoma (**b**) Disease-specific survival of Colorectal Leiomyosarcoma

**Fig. 4:**
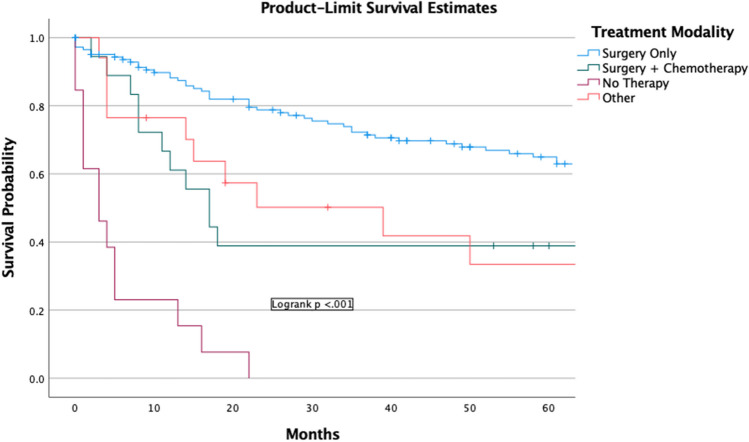
Overall Survival of Different Treatment Modalities. + Other includes Chemotherapy only, Surgery & Radiation, Chemotherapy & Radiation, Combination Therapy (Chemotherapy, Radiation, & Surgery)

The overall 5-year observed survival was 50.3% (95% C.I., 46.3—54.2) (Fig. 3a). The 5-year disease-specific survival (DSS) was 66.1% (95% C.I., 62.0—70.1) (Fig. 3b). The 1-year and 5-year survival with surgery only was 78.7% (95% C.I., 75.1—82.3) and 60.8% (95% C.I., 56.3—65.3), respectively. The survival analysis of chemotherapy only, radiation only, and other therapies not included in this analysis could not be performed due to a limited number of cases. There was a significant difference (p < 0.001) between surgery only, surgery + chemotherapy, no therapy, and other therapies (Fig. 4). Including patients with incomplete information did not change the results of the univariable or multivariable.

### Overall Survival Analysis by Race and Sex

**Fig. 5:**
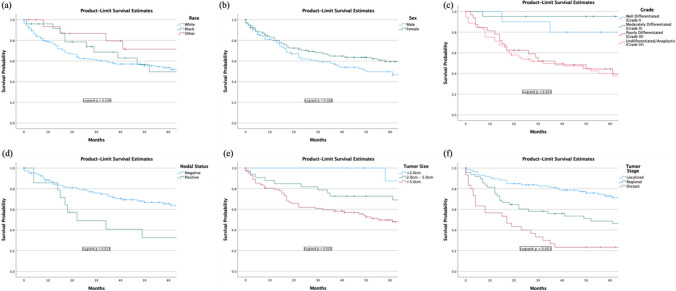
Survival of colorectal leiomyosarcoma by (**a**) race, (**b**) sex, (**c**) grade, (**d**) nodal status, (**e**) tumor size, and (**f**) tumor stage

There was no difference in percent survival rate amongst white, black, and other races (Supplemental Table 1). Race was not associated with survival rates (Fig. 5a).

There was no difference in percent survival rate between male and females (Supplemental Table 2) Sex was not a predictor for survival (Fig. 5b).

### Overall Survival Analysis by Tumor Characteristics

Higher grade was indicated as a negative predictor for survival (p < 0.001) (Fig. 5c). A positive nodal status was considered a negative prognostic factor for survival (p < 0.001) (Fig. 5d). Larger tumor size was indicated as a negative predictor for survival (p < 0.001) (Fig. 5e). A higher tumor stage was also a negative indicator of survival (p < 0.001) (Fig. 5f).

### Univariable and Multivariable Analyses for Overall Survival

Univariable analysis indicated that increased age was a negative predictor (H.R. 1.036 (1.021—1.052), p < 0.001), that higher grade was a negative predictor (H.R. 1.645 (1.252—2.162), p < 0.001), and that a positive nodal status was a negative predictor (H.R. 2.25 (1.16–4.35), p = 0.016). Univariable analysis also revealed a tumor size of > 5 cm was a negative predictor for survival (H.R. 3.145 (1.707—5.793), p < 0.001) and that both regional stage (H.R. 2.02 (1.25–3.24), p = 0.004) and distant stage (H.R. 3.87 (2.31–6.47), p =  < 0.001) were considered negative predictors. Univariate analysis revealed that undergoing surgery alone led to better outcomes (H.R. 0.34 (0.23–0.52) p < 0.001). Whether a patient underwent surgery and chemotherapy did not change the prognosis of survival in a patient (H.R. 1.72 (0.94–3.16), p = 0.079). Multivariable Cox proportional hazards analysis identified older age (H.R. 1.03 (1.01–1.05), p < 0.001), poorly differentiated grade III (H.R. 7.33 (2.35–22.91), p < 0.001), undifferentiated/anaplastic grade IV (H.R. 5.97 (2.04–17.50), p < 0.001), regional stage (H.R. 4.25 (2.20–8.21), p < 0.001), and distant stage (H.R. 6.07 (3.03–12.14), p < 0.001) as negative predictors for survival. Upon multivariate analysis it was determined that the treatment modalities of Surgery only & Surgery + Chemotherapy did not change prognosis of overall survival. Including patients with incomplete information did not change the results of the univariable or multivariable analyses (data not shown) (Table 2).

**Table 2:** Univariable & Multivariable analyses of independent factors influencing overall survival

			**Univariable Analyses**			**Multivariable Analysis**		
		N (%)	HR	95% CI	*p* Value	HR	95% CI	*p* Value
Patient Characteristics								
Age, per year increase		-	1.04	1.02–1.05	** < 0.001**	1.03	1.01–1.05	** < 0.001**
Clinicopathologic Characteristics								
Tumor size	≤ 2 cm	10 (7.9)	Ref	-	-	Ref	-	-
	2–5 cm	33 (26.2)	5.01	00.66–37.9	0.119	10,531.13	0.00–2.64E + 84	0.922
	> 5 cm	83 (65.9)	9.94	1.37–72.02	**0.023**	30,054.37	0.00–7.51E + 84	0.913
Grade	Grade I	10 (8.5)	Ref	-	-	Ref	-	-
	Grade II	21 (17.9)	0.61	0.16–2.27	0.459	2.01	0.51–7.91	0.317
	Grade III	32 (16.8)	2.71	0.92–8.00	0.070	7.33	2.35–22.91	** < 0.001**
	Grade IV	54 (28.3)	3.00	1.07–8.43	**0.037**	5.97	2.04–17.50	** < 0.001**
Node Status	Negative	46 (24.1)	Ref	-	-	-	-	-
	Positive	89 (46.6)	2.25	1.16–4.35	**0.016**	1.95	0.63–6.05	0.247
Disease Stage	Localized	87 (45.5)	Ref	-	-	-	-	-
	Regional	49 (25.7)	2.02	1.25–3.24	**0.004**	4.25	2.20–8.21	** < 0.001**
	Distant	30 (15.7)	3.87	2.31–6.47	** < 0.001**	6.07	3.03–12.14	** < 0.001**
Treatment Characteristics								
Treatment	All Other Treatments	48 (25.1)	Ref	-	**-**	Ref	-	-
	Surgery Only	143 (74.9)	0.34	0.23–0.52	** < 0.001**	0.75	0.17–3.31	0.700
	All Other Treatments	173 (90.6)	Ref	-	**-**	Ref	-	-
	Surgery and Chemotherapy	18 (9.4)	1.72	0.94–3.16	0.079	0.45	0.09–2.30	0.339

*HR* hazards ratio, *CI* confidence interval

^*^Patients with missing information were censored

## Discussion

Our study reports on overall survival, survival by different treatment modalities, and clinicopathological factors associated with CR-LMS. The median age of the patient population was 64 years old, similar to that reported by Miettinen et al. (63 years old) [8]. Race and sex are not predictors of survival. The size of the tumor is a predictor of survival in univariate analysis. On multivariate analysis, grade and stage are independent factors, with higher grade and regional/distant metastatic deposits associated with a worse prognosis. Surgery on univariate analysis is a statistically significant independent factor affecting survival. This finding is concordant with the finding reported by Harshavardhan et al. [9], who reported surgery as a predictor of survival in the univariate analysis only.

Yamamoto et al. found a 5-year survival of 51.6%, similar to our results of 50.3% 5-year overall survival [10]. Lindsay et al. showed the risk of disease progression in colorectal LMS as high as 43% in contrast to those of stomach or small bowel, 25% and 32%, respectively [7]. In our study, the most common site of metastatic disease was the liver, which is concordant to the findings reported by Faraj et al. [11].

Our study shows that CR-LMS is associated with a grave prognosis. Previously, CR-LMS has been anecdotally associated with a worse prognosis compared to other GI sites. Our study indicates that the 5-year survival for CR-LMS is worse when compared to that of other GI-site LMS reported in literature. The risk of recurrence is as high as 39–80% and secondary metastasis reaching 55–71% in combined GI LMS [12].

GISTs and leiomyosarcomas differ in their molecular characteristics, response to treatment, and prognosis. GISTs can often be effectively treated with targeted therapy (TKIs) while leiomyosarcomas are typically resistant to chemotherapy and radiation and have a poorer prognosis. Besides cellular atypia and high mitotic activity, p53 protein expression calculated using IF and flow cytometry was found to be significantly higher (90%) in leiomyosarcomas when compared to leiomyomas (14%) [13]. This is a potential objective parameter that can predict the risk of malignant transformation and overall survival in leiomyosarcomas.

The standard of care is complete surgical excision of the tumor with negative margins and lymph node dissection due to the aggressive nature of the disease. Amelie et al. report a case of recurrence of colorectal LMS that recurred in the mesenteric lymph node [14]. Surgical options include anatomical colectomy, local excision, LAR, and APR based on the site and extent of disease with the goal of achieving negative margins (R0 resection). The decision of an open versus minimally invasive approach depends on the surgeon’s experience and skill set [11]. Ifosfamide or ifosfamide and epirubicin are recommended by NCCN guidelines. However, the efficacy of systemic treatment remains controversial as the majority of LMS are chemo-resistant. Anlotinib is recommended as second-line therapy for advanced soft tissue sarcomas (STS). No specific tumor markers exist for LMS. Cox et al. reported increased uptake of 111In-antimyosin in LMS tissue because of increased permeability, which may have a role as a carrier for therapeutic agents [15].

The genetics of leiomyosarcomas in general are not consistent and have a heterogenous landscape but they overall have low TMB. The genetics of intestinal LMS are not well understood. LMS originates from smooth muscle cells, namely fibroblasts and myofibroblasts, and vascular elements, namely pericytes. The most common mutations that LMS harbors are *TP53* mutations and deletions, deletion of *RB-1*, and mutations in *ATRX* (significant in extrauterine LMS). What makes LMS unique and has therapeutic implications is a high expression of ALT-TERT alterations. This makes it potentially susceptible to combination therapy with irinotecan and p53 activator Eprenetapopt [16]. Next generation sequencing (NGS) and genomic profiling of tumors has allowed for precision medicine and has opened up a world of possibilities. The work by Xianggian et al. used gene expression profiling and immunohistochemistry (IHC) to characterize LMS into three molecular subtypes. Their work has also identified potentially actionable genes with a possible role for FGFR inhibitors, CDK2 inhibitors, mTOR inhibitors, erlotinib, etc. [17]. Lei et al. report a case of colorectal LMS harboring *BRCA2* mutations, successfully treated with PARP inhibitor (Olaparib) [18]. Deficient mismatch repair (dMMR) is rare in sarcomas but has been reported to make these patients candidates for immune checkpoint inhibitor therapy [19].

## Limitations

There are limitations of this study related mainly to the data that was available for analysis from the database. Data concerning margin status, mitotic rates, presence or absence of necrosis, and ulceration was not available. With regards to management, SEER had no data related to the type of surgery (MIS vs open), with or without lymph node dissection. There was also no information on the choice of chemotherapy regimen used. Additionally, we were unable to retrieve details regarding the genomic profiles of these tumors. Clinical factors affecting prognosis such as comorbidities, smoking status data is not available in the SEER database.

## Conclusion

Analysis of data from the SEER database reveals that patients with CR-LMS typically present at a median age of 64 years, with a notable prevalence of advanced stages and poor differentiation. Key factors associated with a poorer prognosis include older age, high tumor grade (III/IV), larger tumor size (> 5 cm), and the presence of distant metastases. Despite the limitations in data regarding treatment modalities and genomic profiling, our findings emphasize that surgical resection remains the most effective treatment approach, with significant improvements in survival rates compared to other therapies. The high rates of disease recurrence and metastasis highlight the need for further research and the development of targeted therapeutic strategies. Future efforts should focus on consolidating data from multiple sources to establish comprehensive evidence-based guidelines and enhance collaborative research to better address this rare malignancy.

## Supplementary Information

Below is the link to the electronic supplementary material.

Supplementary file1 (DOCX 18 KB)

## Data Availability

No datasets were generated or analysed during the current study.

## References

[1] Voltaggio L, Montgomery EA. Gastrointestinal tract spindle cell lesions–just like real estate, it’s all about location. Mod Pathol. 2015;28(Suppl 1):S47–66. 10.1038/modpathol.2014.126. 25560599 10.1038/modpathol.2014.126

[2] Avendano TLM, Flores JAC. Leiomyosarcoma of the rectosigmoid junction, a case report. Radiol Case Rep. 2020;15(10):1887–1890. Published 2020 Aug 14. 10.1016/j.radcr.2020.07.052. 10.1016/j.radcr.2020.07.052 PMC7452061 32904201

[3] Crystal JS, Korderas K, Schwartzberg D, Tizio SC, Zheng M, Parker G. Primary Leiomyosarcoma of the Colon: A Report of Two Cases, Review of the Literature, and Association with Immunosuppression for IBD and Rheumatoid Arthritis. Case Rep Surg. 2018;2018:6824643. Published 2018 Jan 30. 10.1155/2018/6824643. 10.1155/2018/6824643 PMC5892970 29780656

[4] Miettinen M, Furlong M, Sarlomo-Rikala M, Burke A, Sobin LH, Lasota J. Gastrointestinal stromal tumors, intramural leiomyomas, and leiomyosarcomas in the rectum and anus: a clinicopathologic, immunohistochemical, and molecular genetic study of 144 cases. Am J Surg Pathol. 2001;25(9):1121–33. 10.1097/00000478-200109000-00002. 11688571 10.1097/00000478-200109000-00002

[5] Ng EH, Pollock RE, Munsell MF, Atkinson EN, Romsdahl MM. Prognostic factors influencing survival in gastrointestinal leiomyosarcomas. Implications for surgical management and staging. Ann Surg. 1992;215:68–77. 1731651 10.1097/00000658-199201000-00010 PMC1242372

[6] Koczkowska M, Lipska BS, Grzeszewska J, Limon J, Biernat W, Jassem J. Primary leiomyosarcoma of the mesentery in two sisters: clinical and molecular characteristics. Pol J Pathol. 2013;64(1):59–63. 10.5114/pjp.2013.34605. 23625602 10.5114/pjp.2013.34605

[7] Alpert L, Al-Sabti R, Graham RP, et al. Smooth muscle tumors of the gastrointestinal tract: an analysis of prognostic features in 407 cases. Mod Pathol. 2020;33(7):1410–9. 10.1038/s41379-020-0492-5. 32051556 10.1038/s41379-020-0492-5 PMC8405135

[8] Miettinen M, Sarlomo-Rikala M, Sobin LH, Lasota J. Gastrointestinal stromal tumors and leiomyosarcomas in the colon: a clinicopathologic, immunohistochemical, and molecular genetic study of 44 cases. Am J Surg Pathol. 2000;24(10):1339–52. 10.1097/00000478-200010000-00003. 11023095 10.1097/00000478-200010000-00003

[9] Senapathi H, Morada A, Perry M, et al. Prognostic Factors in Gastrointestinal Leiomyosarcomas: An Analysis Using the Surveillance, Epidemiology, and End Results (SEER) Database. Cureus. 2021;13(11):e19447. Published 2021 Nov 10. 10.7759/cureus.19447. 10.7759/cureus.19447 PMC8654067 34926025

[10] Yamamoto H, Handa M, Tobo T, et al. Clinicopathological features of primary leiomyosarcoma of the gastrointestinal tract following recognition of gastrointestinal stromal tumours. Histopathology. 2013;63(2):194–207. 10.1111/his.12159. 23763337 10.1111/his.12159

[11] Faraj W, El-Kehdy J, Nounou GE, et al. Liver resection for metastatic colorectal leiomyosarcoma: a single center experience. J Gastrointest Oncol. 2015;6(5):E70–6. 10.3978/j.issn.2078-6891.2015.044. 26487954 10.3978/j.issn.2078-6891.2015.044 PMC4570914

[12] Nagtegaal ID, Odze RD, Klimstra D, et al. The 2019 WHO classification of tumours of the digestive system. Histopathology. 2020;76(2):182–8. 10.1111/his.13975. 31433515 10.1111/his.13975 PMC7003895

[13] Cai J, Jiang Y, Zhang Y, et al. Quantitation of P53 protein expression in gastrointestinal smooth muscle tumors. Clinicopathological correlation and prognostic significance. Chin Med J. 1995;108:669–73. 8575232

[14] Beauchamp A, Hajjar R, Khullar S, Latour M, Schwenter F, Sebajang H. Mesenteric Lymph Node Recurrence of a Primary Colorectal Leiomyosarcoma. Case Rep Surg. 2020;2020:6935834. Published 2020 Mar 23. 10.1155/2020/6935834. 10.1155/2020/6935834 PMC7125469 32257499

[15] Cox PH, Verweij J, Pillay M, Stoter G, Schonfeld D. Indium 111 antimyosin for the detection of leiomyosarcoma and rhabdomyosarcoma. Eur J Nucl Med. 1988;14(1):50–2. 10.1007/BF00252619. 3383905 10.1007/BF00252619

[16] Macha SJ, Koneru B, Burrow TA, et al. Alternative Lengthening of Telomeres in Cancer Confers a Vulnerability to Reactivation of p53 Function. Cancer Res. 2022;82(18):3345–58. 10.1158/0008-5472.CAN-22-0125. 35947641 10.1158/0008-5472.CAN-22-0125 PMC9566554

[17] Guo X, Jo VY, Mills AM, et al. Clinically Relevant Molecular Subtypes in Leiomyosarcoma. Clin Cancer Res. 2015;21(15):3501–11. 10.1158/1078-0432.CCR-14-3141. 25896974 10.1158/1078-0432.CCR-14-3141 PMC4526352

[18] Korde LA, Somerfield MR, Carey LA, et al. Neoadjuvant Chemotherapy, Endocrine Therapy, and Targeted Therapy for Breast Cancer: ASCO Guideline. J Clin Oncol. 2021;39(13):1485–505. 10.1200/JCO.20.03399. 33507815 10.1200/JCO.20.03399 PMC8274745

[19] Siozopoulou V, Domen A, Zwaenepoel K, et al. Immune Checkpoint Inhibitory Therapy in Sarcomas: Is There Light at the End of the Tunnel?. Cancers (Basel). 2021;13(2):360. Published 2021 Jan 19. 10.3390/cancers13020360. 10.3390/cancers13020360 PMC7835811 33478080

