# Supplementary material for: Colorectal Leiomyosarcoma: Demographics Patterns, Treatment Characteristics, and Survival Analysis in the U.S. Population
Source: J Gastrointest Cancer. 2024 Aug 27;55(4):1588–97. doi: 10.1007/s12029-024-01110-x (PMC11464608; doi:10.1007/s12029-024-01110-x)
Supplement: Supplementary file 1 — Supplementary file1 (DOCX 18 KB) [file 12029_2024_1110_MOESM1_ESM.docx]

**Supplemental Table 1:** Univariable & Multivariable analyses of independent factors influencing disease specific survival.

|  | |  | **Univariable Analyses** | | | | | **Multivariable Analysis** | | |
| --- | --- | --- | --- | --- | --- | --- | --- | --- | --- | --- |
|  |  | N (%) | | HR | 95% CI | *p* Value | HR | | 95% CI | *p* Value |
| Patient Characteristics | | | | | | | | | | |
| Age, per year increase | | - | | 1.03 | 1.01-1.05 | **<0.001** | 1.04 | | 0.99-1.093 | 0.166 |
| Clinicopathologic Characteristics | | | | | | | | | | |
| Tumor size | ≤2 cm | 10 (7.9) | | Ref | - | - | Ref | | - | - |
|  | 2-5 cm | 33 (26.2) | | 2.00E4 | 0.00-1.6E71 | 0.900 | 1.02E4 | | 0.00-8.14E1000 | 0.935 |
|  | >5 cm | 83 (65.9) | | 3.10E4 | 0.00-2.45E71 | 0.895 | 2.67E4 | | 0.00-2.11E101 | 0.929 |
| Grade | Grade I | 10 (8.5) | | Ref | - | - | Ref | | - | - |
|  | Grade II | 21 (17.9) | | 0.23 | 0.02-2.55 | 0.231 | 0.55 | | 0.05-6.20 | 0.626 |
|  | Grade III | 32 (16.8) | | 3.24 | 0.74-14.3 | 0.120 | 6.41 | | 1.42-29.9 | **0.016** |
|  | Grade IV | 54 (28.3) | | 3.32 | 0.78-14.1 | 0.104 | 5.105 | | 1.17-22.38 | **0.031** |
| Node Status | Negative | 46 (24.1) | | Ref | - | - | - | | - | - |
|  | Positive | 89 (46.6) | | 1.31 | 0.46-3.75 | 0.613 | 0.63 | | 0.12-3.32 | 0.589 |
| Disease Stage | Localized | 87 (45.5) | | Ref | - | - | - | | - | - |
|  | Regional | 49 (25.7) | | 3.60 | 1.80-7.20 | **<0.001** | 4.37 | | 1.80-10.62 | **0.001** |
|  | Distant | 30 (15.7) | | 6.59 | 3.15-13.8 | **<0.001** | 7.27 | | 2.83-18.65 | **<0.001** |
| Treatment Characteristics | | | | | | | | | | |
| Treatment | All Other Treatments | 48 (25.1) | | Ref | - | **-** | Ref | | - | - |
|  | Surgery Only | 143 (74.9) | | 0.31 | 0.18-0.55 | **<0.001** | 0.73 | | 0.16-3.34 | 0.685 |
|  | All Other Treatments | 173 (90.6) | | Ref | - | **-** | Ref | | - | - |
|  | Surgery and Chemotherapy | 18 (9.4) | | 1.22 | 0.49-3.08 | 0.669 | 0.22 | | 0.03-1.70 | 0.145 |
| HR = hazards ratio; CI = confidence interval | | | | | | | | | | |

*Associated with worse survival outcomes

^+^Associated with better survival outcomes

**Supplemental Table 1:** Race Survival Analysis

| **Survival** | **Percent Survival Rate (%, C.I. 95%)** | | |
| --- | --- | --- | --- |
|  | **White** | **Black*** | **Other*** |
| 1 year | 62.2% (58.1-66.3) | 78.0% (69.3-86.7) | 86.6% (78.1-95.5) |
| 5 years | 47.9% (43.5-52.3) | 50.2% (38.6-61.8) | 71.6% (59.4-83.8) |

*All other races

**Supplemental Table 2:** Sex Survival Analysis

| **Survival** | **Percent Survival Rate (%, C.I. 95%)** | |
| --- | --- | --- |
|  | **Male** | **Female** |
| 1 year | 61.8% (56.4-67.2) | 70.2% (65.6-74.8) |
| 5 years | 44.7% (38.9-50.5) | 55.0% (49.6-60.4) |
